# Influence of Reinforcement Bars on Concrete Pore Structure and Compressive Strength

**DOI:** 10.3390/ma13030658

**Published:** 2020-02-01

**Authors:** Jianmin Hua, Fengbin Zhou, Lepeng Huang, Zengshun Chen, Yemeng Xu, Zhuolin Xie

**Affiliations:** 1 School of Civil Engineering, Chongqing University, Chongqing 400045, China; huajianmin@cqu.edu.cn (J.H.); 20141613158@cqu.edu.cn (F.Z.); zengshunchen@cqu.edu.cn (Z.C.); xu.ym@cqu.edu.cn (Y.X.); 20151613111@cqu.edu.cn (Z.X.); 2 Key Laboratory of New Technology for Construction of Cities in Mountain Area (Chongqing University), Ministry of Education, Chongqing 400045, China

**Keywords:** reinforcement bar, pore structure, concrete strength, strength prediction

## Abstract

In this research, the influence of reinforcement bars on concrete pore structure and compressive strength was experimentally investigated. Concrete samples with two mixture ratios and nine reinforcement ratios were provided. Tests were conducted on concrete pore structure and compressive strength at three ages (3 d, 7 d, and 28 d). It was found that reinforcement bars changed the concrete pore structure. In terms of size, the pore structure of concrete increased with the increase of reinforcement ratio. At the same age, concrete compressive strength in reinforced concrete specimens saw a gradual reduction when reinforcement ratio increased. A formula was proposed to calculate the compressive strength of concrete in reinforced specimens according to the strength of unreinforced concrete.

## 1. Introduction

Concrete is a cement-based material, whose strength is affected by pores and microcracks, especially under the action of thermal loads [1,2,3,4,5]. Extensive research was carried out to probe into the relationship between concrete pore structure and compressive strength. Li et al. [6] studied the connection between concrete strength and pore structure by introducing an influencing coefficient of pore size. Huang et al. [7] established an expression to calculate concrete compressive strength according to the relative specific surface area of pore structure. Lian et al. [8] investigated the association between concrete pore structure and compressive strength. Gao et al. [9] prepared defective specimens by adding air-entraining agents to concrete, and studied the influence of pore structure on concrete strength. It was generally found that concrete strength was higher for a denser pore structure [6,7,8,9,10,11,12].

In engineering practices, concrete is often combined with reinforcement bars to form a reinforced concrete structure. The existing studies showed that there are interfacial transition zones (ITZ) in reinforced concrete structures between reinforcement bar and concrete. The porosity of concrete in ITZ area is higher than that in non ITZ area [13], thereby leading to changes in a series of properties including concrete strength [14,15,16], especially beneath the horizontal reinforcement and in some places where the reinforcement ratio is high and the concrete cannot be well vibrated. In these areas, the porosity of ITZ is further improved, which further degrades the property of concrete [15,17,18]. Meanwhile, when water is gradually consumed by cement hydration and dries in concrete, the capillary stress generated on concrete pores continuously decreases the spacing between concrete particles and causes the volume of concrete to decrease on a macro scale (this phenomenon is called shrinkage). Because of the restraint effects of reinforcement bars, restraint stress is generated in the opposite direction of shrinkage inside concrete and decreases concrete shrinkage. Compared with plain concrete, reinforced concrete with a reinforcement ratio of 1 to 7% saw a drop of 30 to 80% in shrinkage after 28 d [19,20,21,22,23,24]. The pore structure of concrete will be changed by the restraints in its deformation. Previous studies [24,25] revealed that concrete with lateral restraints experienced a significant decline in the values of different pore structure parameters (porosity and average pore size) than that without lateral restraints. However, the effect of restricting the reduction of concrete volume (shrinkage for example) on concrete pore structure remains unknown.

Based on the previous findings about the connection between concrete strength and porosity and changes in concrete pore structure by restraints, it could be reasonably inferred that the pore structure of reinforced concrete would be greater than that of unreinforced concrete in the shrinkage process of concrete because of the restraint effect of reinforcement, which caused the strength of reinforced concrete to be lower than that of unreinforced concrete. However, this reference remains to be confirmed by research.

In the early phase of construction, it is necessary to carry loads by scaffolding because of low concrete strength. According to Chinese standards [26], the removal of scaffolding during construction depends on concrete compressive strength. Scaffolding is allowed to be removed when the concrete compressive strength of general components and other important components reaches 75 and 100% of design strength respectively. Because of several limitations in engineering practices, the decision of whether concrete compressive strength meets requirements is made by testing concrete blocks that are not restrained by reinforcement bars [26]. Therefore, consideration is not given to the possible influence of reinforcement bars on concrete strength and pore structure. The existing evaluation methods of concrete strength at the construction site may increase the possibility of failure in building structure during construction if research suggested that reinforcement bars would change concrete pore structure and then reduce concrete compressive strength during the shrinkage of concrete.

This research experimentally investigated the influence of reinforcement bars on concrete pore structure and compressive strength. Concrete samples with two mixture ratios and nine reinforcement ratios (0–6.56%) were given. At different ages (3 d, 7 d, and 28 d), the mercury intrusion method was adopted to test the pore structure of concrete samples with two mixture ratios and a reinforcement ratio of 0%, 1.14%, 3.24%, and 6.56%. Moreover, the compressive strength of all types of samples was also tested. All specimens were well vibrated before testing. A comparison was made between concrete pore structure and compressive strength with different reinforcement ratios to study the changes in concrete pore structure and compressive strength after reinforcement.

## 2. Experiments

### 2.1. Materials

Two different concrete mixing ratios were applied in this study (Table 1). Cement and fly ash were chosen as cementing materials, whose chemical and physical properties are shown in Table 2. Quartz sand was used as fine aggregate, with a specific gravity of 2.8g/cm^3^, a fineness modulus of 3.0, and a particle size distribution of 0.2–4 mm. Crushed limestone was used as coarse aggregate with a specific gravity of 2.8 g/cm^3^ and a particle size range of 5–20 mm. The superplasticizer used is polycarboxylate superplasticizer with a water-reducing rate of 30–35%. The information of reinforcement bars used in this study is shown in Table 3.

### 2.2. Specimens for Concrete Strength Tests

**Figure 1:**
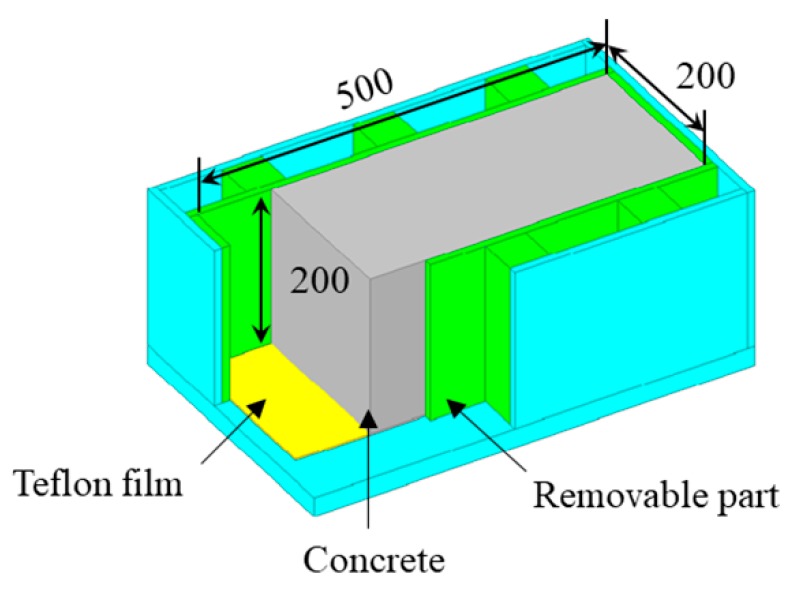
Teflon mold for forming specimens (All dimensions are in mm).

Produced by a Teflon mold, specimens whose dimension is 200 mm × 200 mm × 500 mm were adopted in the present study (as shown in Figure 1). The four sides of the mold were removed after initial concrete setting (in green in Figure 1), and its bottom was laid with two layers of 1-mm thick Teflon film (in yellow in Figure 1). With these measures, concrete specimens were only subject to reinforcement bars.

**Figure 2:**
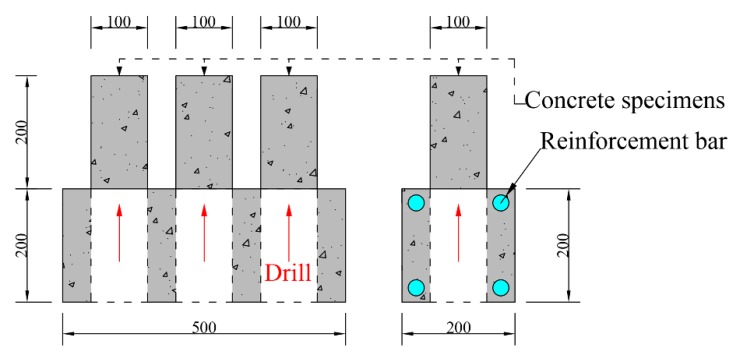
Specimen for measuring the pore structure and compressive strength of concrete (All dimensions are in mm).

Eighteen kinds of concrete specimens that owned two mixture ratios and nine reinforcement ratios (ρ = 0%, 1.14%, 1.56%, 2.05%, 2.61%, 3.24%, 3.95%, 5.16%, and 6.56%) were used in the present investigation (Table 4). Reinforcement bars of different diameters were evenly put in four corners of concrete cross-section (Figure 2). For each type, three specimens were prepared to test concrete compressive strength at three ages. All specimens were cured and tested in a constant environment (20 ± 2 °C, 65 ± 5% RH).

After 3 d, 7 d, and 28 d, three cylindrical samples whose diameter is 100 mm and height is 200 mm (height/diameter = 2) were drilled from each type of specimen to measure the concrete compressive strength (according to the JGJ/T384 standard [27]) (Figure 2). The test method of concrete strength was adopted according to Chinese national standard GB/T50081 [28].

### 2.3. Test Specimens for Concrete Pore Structure

Concrete pore structure with a reinforcement ratio of 0%, 1.14%, 3.24%, and 6.56% (C30-R0, C30-R1, C30-R5, C30-R8 and C60-R0, C60-R1, C60-R5, C60-R8) was tested. The remaining specimens after drilling were used as the samples for concrete porosity tests. The concrete samples were first crushed and then filtered by a sieve to collect concrete particles with a size of 2.5–5 mm. Acetone was applied to prevent concrete hydration, and a vacuum dryer was used to dry the samples. At last, concrete pore structure was tested by an automatic mercury porosimeter that has an aperture measurement range of 0.003–1000 μm, low-pressure analysis pressure range of 0–345 kPa, high-pressure analysis pressure range of 101.325–414000 kPa, and a resolution ratio of 69 Pa.

## 3. Experimental Results and Discussion

### 3.1. Change of Pore Structure

Table 5 presents the pore structure parameters of concrete specimens with a reinforcement ratio of 0%, 1.14%, 3.24%, and 6.56% at different ages, and shows that the size of concrete pore structure gradually declined with curing age under the same mixing and reinforcement ratios. For instance, the average and median pore diameters, critical diameter, and porosity of C30-R0 concrete after 3 d were 26.48 nm, 83.65 nm, 82.73 nm, and 24.31% respectively, which are 8.97 nm, 57.09 nm, 57.1 nm, and 6.66% higher than those after 28 d respectively. Moreover, the proportion of pores whose diameter is below 50 nm at 28 d was noticeably higher than that at 3 d in C30-R0 concrete. Similar patterns were also found in other specimens. The size of concrete pore structure gradually decreased with curing age for two main reasons, namely the filling of pores by hydrated products and the decline of the size between a variety of pore walls and microcracks in concrete because of capillary stress [29,30,31,32].

It was also observed that the placement of reinforcement bars led to a significant change in concrete pore structure whose size saw a great increase when reinforcement ratio increased. For example, the critical diameter of C60-R8 concrete with a reinforcement ratio of 6.56% was 44.30 nm at 28 d, which is higher than those of C60-R5 (ρ = 3.24%), C60-R1 (ρ = 1.14%), and C60-R0 (ρ = 0%) specimens (36.85 nm, 27.33 nm, and 20.11 nm respectively). Similar trends were also observed in C30 concrete specimens, other ages, and pore structure parameters.

**Figure 3:**
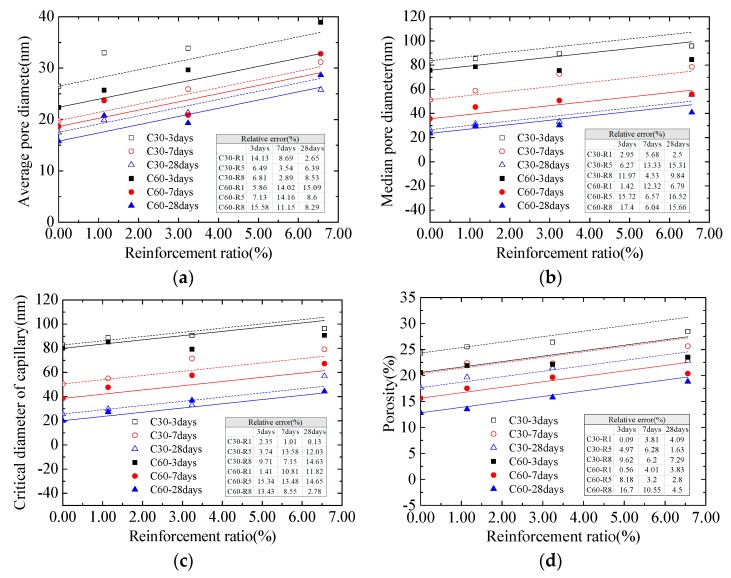
Connection between concrete pore structure and reinforcement ratio: (**a**) average pore diameter; (**b**) median pore diameter; (**c**) critical diameter of capillary; (**d**) porosity.

Figure 3 displays the connection between the reinforcement ratio of concrete and different parameters of pore structure (average and median pore diameters, critical diameter as well as porosity). Notably, the size of concrete pore structure had a linear relationship with reinforcement ratio. Therefore, the change of concrete pore structure caused by reinforcement bars could be expressed by the following equation:

*S_ρ_* = *S*_0_ + *nρ* (1)

where *S_ρ_* is the parameter of concrete pore structure corresponding to the reinforcement ratio *ρ*; *S*_0_ represents the parameter of concrete pore structure corresponding to the reinforcement ratio of 0%; *n* is the increase in the parameters of pore structure for each unit of increase in reinforcement ratio.

Regression analysis was made on obtained testing data to get the value of *n* (Table 6). Figure 3 expresses the relative errors between the pore structure parameters of concrete calculated by Equation (1) and the measured values. It was observed that the calculated values well agreed with the measured ones.

**Figure 4:**
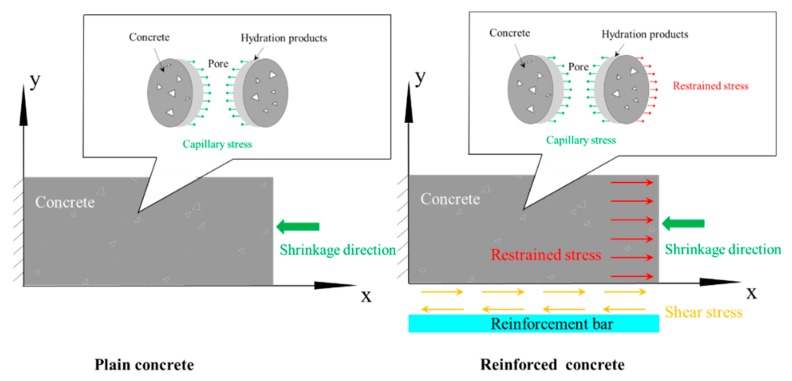
Changes in concrete pore structure.

Concrete is a kind of porous material. As water is consumed by hydration and dries in concrete pores, the capillary stress generated on the pore walls of concrete results in concrete shrinkage. In plain concrete specimens (without reinforcement bars), a decline appeared in concrete pore structure because of filling by hydrated products and capillary stress, as shown in Figure 4. Restrictions of reinforcement bars in the free shrinkage of concrete would lead to the occurrence of shear stress on the interface between concrete and reinforcement bars, and generated normal stress (known as restraint stress) in concrete in a direction opposite to the shrinkage of concrete caused by capillary stress [23] (Figure 4). The equation below can be used to calculate restraint stress [23]:

(2) $$\sigma_{t}=\frac{\varepsilon_{sh}\cdot E_{c}}{1+\frac{E_{c}}{\rho E_{s}}}$$

where *E_c_* and *E_s_* are the elastic moduli of concrete and reinforcement bars respectively; *ε_sh_* represents the free shrinkage of concrete.

From Equation (2), it can be seen that restraint stress saw an increase when reinforcement ratio increased, thereby reducing concrete pore structure because of capillary stress. Therefore, the experimental results indicated that the pore structure of concrete increased when reinforcement ratio increased.

### 3.2. Concrete Compressive Strength

Table 7 presents concrete compressive strength at different ages. It was noticeable that the compressive strength of concrete increased with curing age for each specimen. In each group, concrete compressive strength after 3 d reached 56 to 65% of that after 28 d, whereas concrete compressive strength after 7 d reached 79 to 91% of that after 28 d. Obviously, concrete strength was characterized by a rapid increase in the early age (3–7 d) followed by a gradual decrease in growth rate.

However, compressive strength gradually decreased when reinforcement ratio increase in specimens, which are the same in strength but different in reinforcement ratio. For specimens with the same strength grade, the highest compressive strength was always obtained in specimens whose reinforcement ratio is 0%. For example, the compressive strength of C30-R8 (ρ = 6.56%) at 28 d was 24.6 MPa, which is lower than that of C30-R7 (ρ = 5.16%), C30-R6 (ρ = 3.95%), C30-R5 (ρ = 3.24%), C30-R4 (ρ = 2.61%), C30-R3 (ρ = 2.05%), C30-R2 (ρ = 1.56%), C30-R1 (ρ = 1.14%), and C30-R0 (ρ = 0%) (25.2, 26.6, 27.5, 28.4, 30.3, 31.5, 33.2, and 34.5 MPa respectively).

**Figure 5:**
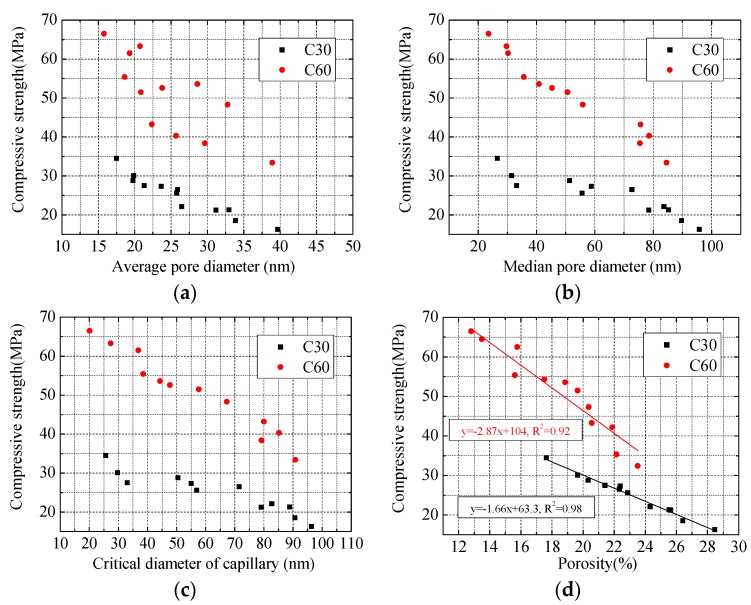
Relationship between compressive strength and parameters of pore structure: (**a**) average pore diameter; (**b**) median pore diameter; (**c**) critical diameter; (**d**) porosity.

Figure 5 presents the connection between the strength and pore parameters of specimens with a reinforcement ratio of 0%, 1.14%, 3.24%, and 6.56%. For concrete specimens of the same strength grade, concrete compressive strength decreased in the whole when pore structure parameters increased, which was consistent with the conclusion that concrete with denser pore structure would have higher strength. In restrained specimens, the existence of constrained tensile stress reduced the effect of capillary pore stress on pore structure, which caused the pore structure of restrained specimens to be larger than that of plain concrete specimens. In restrained specimens, restraint effect would be stronger and concrete pore structure would be larger when reinforcement ratio was higher. Therefore, it was observed that concrete strength decreased when reinforcement ratio increased in tests.

The relationship between concrete compressive strength and pore structure (mainly porosity) could be described ideally in a linear, exponential, or polynomial form [10,11,12]. In Figure 5d, the relationship between concrete porosity and compressive strength is fitted by a linear function, which shows that the linear fitting effect of concrete with the same strength grade is better, but the strength of concrete with different strength grades varied greatly when porosity was similar. For instance, the compressive strength of C30 specimen was 27.5 MPa when its porosity was 21.4%, while the compressive strength of C60 specimen was 40.3 MPa when its porosity was 21.88%, which was 12.8 MPa higher than that of C30. Similar phenomena were also found in studies by Li [33] and Gao [34].

**Figure 6:**
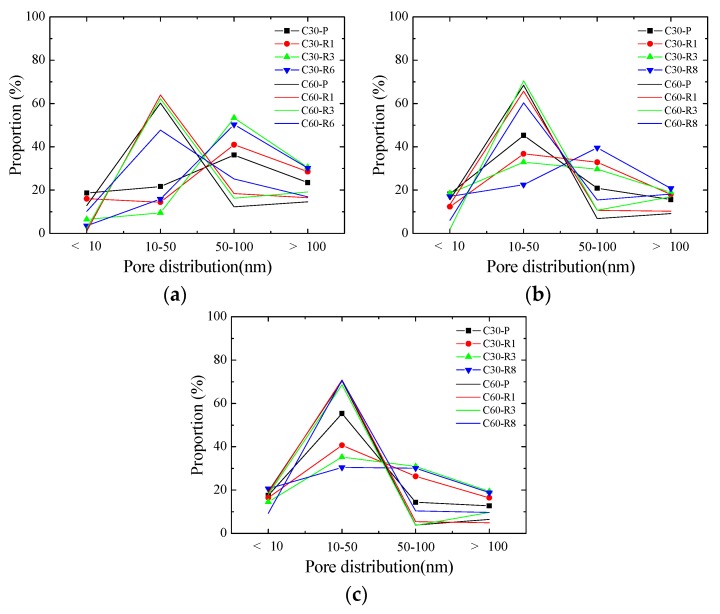
Comparison of pore size distribution between C30 specimen and C60 specimen at three ages: (**a**) 3 d; (**b**) 7 d; (**c**) 28 d.

Figure 6 shows the comparison of pore size distribution between C30 and C60 at 3 d, 7 d, and 28 d, which demonstrated significant differences in their pore size distribution. At each age, the pore diameters of C30 and C60 were mainly distributed between 50–100 nm and 10–50 nm respectively. The porosity of concrete reflected the content of all pores, and different pore size distribution might also lead to the same or similar porosity [33,34]. Meanwhile, previous studies suggested that the compressive strength of concrete would be higher when a small-aperture pore in concrete took up a larger proportion, which thus led to the difference in the strength of C30 and C60 when porosity was similar in tests.

### 3.3. Relationship between the Compressive Strength and Reinforcement Ratio of Concrete

This study showed that reinforcement bars would reduce the decline of concrete pore structure caused by capillary stress, thereby causing reinforced specimens to be inferior to unreinforced ones in compressive strength. Concrete compressive strength gradually decreased when reinforcement ratio increased. In engineering practices, the risk of accidents may be increased if the removal of scaffolding is determined by the compressive strength of unreinforced concrete.

It is difficult to directly test the pore structure parameters of concrete at the construction site. Therefore, the connection between the reinforcement ratio and compressive strength of concrete is established in Equation (3), which can be used to evaluate the strength of reinforced concrete according to the known strength of unreinforced concrete.

*f_cρ_* = *f*_*c*0_*exp^bρ^* (3)

where *f_cρ_* and *f*_*c*0_ are the compressive strength of concrete with a reinforcement ratio of *ρ* and 0% respectively; *b* refers to a calculation parameter and is equal to 5.1 in this study.

**Figure 7:**
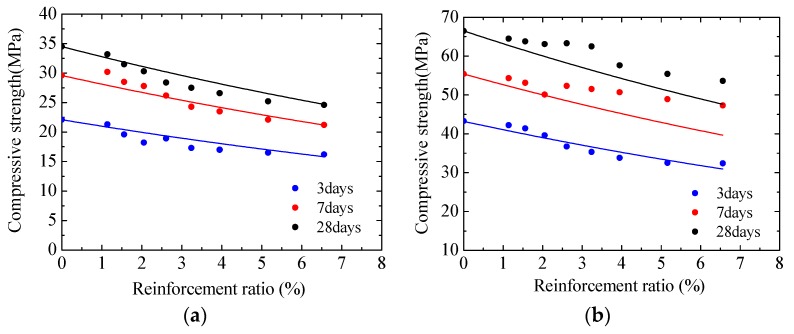
Comparison of predicted and test results: (**a**) C30 concrete (**b**) C60 concrete.

Figure 7 shows concrete compressive strength predicted by using Equation (3) under the condition of different reinforcement ratios. At 3 d, 7 d, and 28 d, the average prediction errors of C30 were 4.73%, 3.26%, and 3.75% respectively and the maximum error was 9.37%, while the average prediction errors of C60 were 3.35%, 7.96%, and 6.74% respectively and the maximum error was 16.17%. As shown in Figure 6, the predicted results well agreed with the actual test values. It should be noted that the parameter b may also be affected by other conditions, such as the additives used in concrete, the age of concrete, and the external environment. In the future, it will be necessary to carry out further experiments to discuss this issue.

## 4. Conclusions

The present study experimentally investigated the effects of reinforcement bars on concrete pore structure and compressive strength that were tested at three ages in the case of various reinforcement ratios. The main observations are presented below:

(1) The size of concrete pore structure gradually decreased during cement hydration because of the filling of hydrated products and capillary stress. The proportion of pores whose diameter ranges from 0 to 50 nm increased, whereas that of pores whose diameter is above 50 nm declined noticeably.
(2) The size of concrete pore structure gradually increased when reinforcement ratio increased. A linear relationship was found between pore structure parameters (average and media pore diameters, critical diameter and porosity) and reinforcement ratio.
(3) At the same age, the compressive strength of reinforced concrete specimens was inferior to that of unreinforced ones and gradually declined with the increasing reinforcement ratio.
(4) In engineering practices, the risk of accidents may be increased if the removal of scaffolding is determined by the compressive strength of unreinforced concrete. A formula was proposed in the present study to forecast the strength of reinforced concrete using unreinforced concrete strength and reinforcement ratio. The predicted results well agreed with the measured ones.

## Figures and Tables

**Table 1:** Mixing proportion (relative weight ratios to cement).

Mix	Cement	Water	Fly Ash	Sand	Coarse Aggregate	Superplasticizer
C30	1.00	0.5	0.26	2.36	2.71	0.03
C60	1.00	0.34	0.26	1.64	2.46	0.03

**Table 2:** Chemical and physical information of cement and fly ash.

Composition % (mass)	Cement	Fly Ash
SiO_2_	22.07	50.22
CaO	64.74	4.52
Al_2_O_3_	5.44	21.33
Fe_2_O_3_	4.18	14.01
MgO	1.39	1.14
SO_3_	0.54	1.39
Specific surface (cm^2^/g)	6465	4598
Density (g/cm^3^)	3.13	2.21

**Table 3:** Properties of reinforcement bar.

Restraint	Elastic Modulus (×10^4^ MPa)	Yield Strength (MPa)	Ultimate Strength (MPa)
Reinforcement bar	20.6	400	580

**Table 4:** Information of specimens.

No.	Strength	Reinforcement Ratio (%)	Reinforcement Bars Diameter (mm)
C30-R0	C30	0	-
C30-R1		1.14	12
C30-R2		1.56	14
C30-R3		2.05	16
C30-R4		2.61	18
C30-R5		3.24	20
C30-R6		3.95	22
C30-R7		5.16	25
C30-R8		6.56	28
C60-R0	C60	0	-
C60-R1		1.14	12
C60-R2		1.56	14
C60-R3		2.05	16
C60-R4		2.61	18
C60-R5		3.24	20
C60-R6		3.95	22
C60-R7		5.16	25
C60-R8		6.56	28

**Table 5:** Pore parameters of concrete at different ages.

NO.		Average Pore Diameter (nm)	Median Pore Diameter (nm)	Critical Diameter (nm)	Porosity (%)	Pore Distribution (%)			
						<10 nm	10–50 nm	50–100 nm	>100 nm
C30-R0	3	26.48	83.65	82.73	24.31	18.66	21.66	36.23	23.45
	7	19.78	51.34	50.49	20.33	18.19	45.29	20.87	15.65
	28	17.51	26.56	25.63	17.65	17.58	55.33	14.32	12.77
C30-R1	3	32.96	85.24	88.81	25.53	16.06	14.45	40.96	28.53
	7	23.66	58.78	55.03	22.38	12.35	36.77	32.87	18.01
	28	19.86	31.45	29.66	19.65	16.52	40.68	26.34	16.46
C30-R5	3	33.86	89.69	90.68	26.4	6.52	9.56	53.37	30.55
	7	25.88	72.69	71.54	22.33	18.51	32.89	29.66	18.94
	28	21.33	33.15	33.00	21.4	14.52	35.23	30.89	19.36
C30-R8	3	39.68	95.80	96.34	28.46	3.58	15.88	50.28	30.26
	7	31.18	78.51	79.11	25.63	17.07	22.54	39.54	20.85
	28	25.80	55.65	56.92	22.87	20.64	30.45	30.12	18.79
C60-R0	3	22.36	75.66	79.99	20.56	12.88	60.23	12.29	14.60
	7	18.63	35.64	38.54	15.62	15.52	68.44	6.86	9.18
	28	15.79	23.56	20.11	12.81	19.29	70.41	3.84	6.46
C60-R1	3	25.69	78.65	85.18	21.88	1.07	63.98	18.44	16.51
	7	23.79	45.33	47.68	17.52	13.31	65.66	10.72	10.31
	28	20.74	29.68	27.33	13.49	18.86	70.80	5.45	4.89
C60-R5	3	29.66	75.46	79.18	22.15	2.36	62.19	16.28	19.17
	7	20.86	50.63	57.65	19.65	1.86	70.47	10.78	16.89
	28	19.31	30.23	36.85	15.77	18.01	68.59	3.71	9.69
C60-R8	3	38.92	84.56	90.76	23.52	10.20	47.72	25.13	16.94
	7	32.78	55.88	67.25	20.36	6.05	60.35	15.43	18.17
	28	28.66	40.79	44.30	18.85	9.30	70.65	10.36	9.69

**Table 6:** Parameters n for different pore parameters.

Coefficient	Average Pore Diameter (nm)	Median Pore Diameter (nm)	Critical Diameter (nm)	Porosity
*n*	160	360	350	105

**Table 7:** Test results of concrete strength (MPa).

NO.	Strength (Standard Deviation)			NO.	Strength (Standard Deviation)		
	3 d	7 d	28 d		3 d	7 d	28 d
C30-R0	22.1(1.05)	29.6(0.12)	34.5(1.10)	C60-R0	43.3(2.05)	55.4(1.46)	66.5(2.14)
C30-R1	21.3(1.20)	30.2(1.30)	33.2(0.15)	C60-R1	42.2(1.20)	54.3(0.87)	64.5(0.91)
C30-R2	19.6(0.25)	28.5(0.25)	31.5(1.25)	C60-R2	41.4(0.20)	53.1(1.10)	63.8(1.32)
C30-R3	18.2(1.15)	27.8(0.31)	30.3(0.15)	C60-R3	39.6(2.20)	50.1(1.79)	63.1(0.15)
C30-R4	18.9(0.95)	26.2(1.15)	28.4(1.20)	C60-R4	36.7(0.35)	52.3(1.52)	63.3(0.17)
C30-R5	17.3(1.20)	24.3(0.06)	27.5(1.21)	C60-R5	35.3(1.15)	51.5(0.96)	62.5(1.50)
C30-R6	17.0(0.21)	23.5(1.25)	26.6(0.15)	C60-R6	33.8(0.40)	50.7(1.60)	57.6(0.2)
C30-R7	16.5(1.30)	22.1(0.26)	25.2(1.35)	C60-R7	32.5(0.32)	48.9(1.05)	55.4(1.07)
C30-R8	16.2(0.20)	21.2(0.31)	24.6(0.25)	C60-R8	32.4(1.05)	47.3(0.25)	53.6(0.65)
